# Transbronchial Cryobiopsy Compared to Forceps Biopsy for Diagnosis of Acute Cellular Rejection in Lung Transplants: Analysis of 63 Consecutive Procedures

**DOI:** 10.3390/life12060898

**Published:** 2022-06-15

**Authors:** Carolin Steinack, Ariana Gaspert, Fiorenza Gautschi, René Hage, Bart Vrugt, Alex Soltermann, Macé Matthew Schuurmans, Daniel Franzen

**Affiliations:** 1 Department of Pulmonology, Center of Lung Transplantation, Center of Adult Cystic Fibrosis, Interventional Lung Center, University Hospital Zurich, 8091 Zurich, Switzerland; fiorenza.gautschi@usz.ch (F.G.); rene.hage@usz.ch (R.H.); mace.schuurmans@usz.ch (M.M.S.); daniel.franzen@spitaluster.ch (D.F.); 2 Faculty of Medicine, University of Zurich, 8006 Zurich, Switzerland; ariana.gaspert@usz.ch; 3 Department of Pathology and Molecular Pathology, University Hospital Zurich, 8091 Zurich, Switzerland; 4 Department of Pathology, Cantonal Hospital Münsterlingen, 8596 Münsterlingen, Switzerland; bart.vrugt@stgag.ch; 5 Pathology Länggasse, 3063 Ittigen, Switzerland; alex.soltermann@patholaenggasse.ch

**Keywords:** cryobiopsy, acute rejection, lung transplantation

## Abstract

Background: Acute cellular rejection (ACR) is a complication after lung transplantation (LTx). The diagnosis of ACR is based on histologic findings using transbronchial forceps biopsy (FB). However, its diagnostic accuracy is limited because of the small biopsy size and crush artifacts. Transbronchial cryobiopsy (CB) provides a larger tissue size compared with FB. Methods: FB and CB were obtained consecutively during the same bronchoscopy (February 2020–April 2021). All biopsies were scored according to the ISHLT criteria by three pathologists. Interobserver agreement was scored by the kappa index. We assessed the severity of bleeding and the presence of pneumothorax. Results: In total, 35 lung transplant recipients were included, and 126 CBs and 315 FBs were performed in 63 consecutive bronchoscopies. ACR (A1–A3, minimal–moderate) was detected in 18 cases (28.6%) by CB, whereas ACR was detected in 3 cases (4.8%) by FB. Moderate and severe bleeding complicated FB and CB procedures in 23 cases (36.5%) and 1 case (1.6%), respectively. Pneumothorax occurred in 6.3% of patients. The interobserver agreement was comparable for both CB and FB. Conclusions: CB provided an improved diagnostic yield for ACR diagnosis, leading to reclassification and changes in treatment strategies in 28.6% of cases. Prospective studies should better define the role of CB after LTx.

## 1. Introduction

Lung transplantation (LTx) is a life-saving therapeutic option in selected patients with end-stage pulmonary disease [1]. Chronic obstructive pulmonary disease (COPD), interstitial lung disease (ILD), and cystic fibrosis (CF) are the most common indications for LTx according to the recent Thoracic Transplant Registry Report of the International Society for Heart and Lung Transplantation (ISHLT) [1]. Acute cellular rejection (ACR) is detected in 27% of lung transplant recipients (LTRs) within the first year after LTx and is an important risk factor for chronic allograft dysfunction (CLAD) and mortality [1,2,3]. Allograft dysfunction is the most common cause of death following LTx [1].

CLAD is defined as a substantial loss of lung function after other factors, particularly infections, have been excluded [2]. Readily accessible diagnostic procedures to detect ACR at the earliest possible occasion are crucial for post-transplant survival [2,3,4]. Bronchoalveolar lavage (BAL) is a widely accepted method for identifying an underlying infectious disease, and a differential cellularity profile in BAL might raise the suspicion of the presence of acute ACR [5]. For example, lymphocytosis > 20%, neutrophilia ≥ 12% without microbiological evidence for infections, and the presence of eosinophils and basophils in BAL might provide hints for the presence of acute ACR [5,6]. Research on novel biomarkers in BAL fluid is currently underway [7,8]. However, histopathological confirmation is still indispensable to establish a diagnosis of ACR. The current gold standard for diagnosis and grading of ACR in order to optimize the immunosuppressive strategy is a histological diagnosis based on transbronchial biopsy (TBB) specimens obtained by forceps biopsy (FB), whereby a minimum of five samples is required [9]. FB has a reasonable risk profile in experienced centers. However, FB is frequently associated with sampling errors because of small sample sizes and crush artifacts and is a relevant source of interobserver variability concerning histologic interpretation [10]. Another approach for obtaining adequate lung tissue is surgical lung biopsy. However, this is not justified because of the increased risk of complications associated with immunosuppression, such as infections and impaired wound healing.

Given these limitations concerning the limited diagnostic yield of FB and unjustifiable risks associated with surgical lung biopsy, transbronchial cryobiopsy (CB) has arisen as a promising alternative diagnostic procedure [11,12,13,14,15,16,17,18,19,20]. It has been used in bronchoscopic procedures since the 1970s and was recently implemented for the diagnosis of several pulmonary diseases, such as ILD, pulmonary infections, and malignancies [11,12]. Its procedural and diagnostic safety profiles have been demonstrated in several randomized trials and cohorts [13,14,15,16,17,18,19,20]. The value of CB for obtaining a conclusive diagnosis of ACR is discussed controversially because of the lack of safety and efficacy data in LTRs [21,22,23,24,25,26,27].

This study aimed to provide data on the safety, diagnostic utility, and impact on treatment decisions after CB in LTRs. We further sought to investigate the interobserver agreement of the histopathological results reported by three independent lung transplant pathologists. To our knowledge, this is the first study to investigate consecutive CBs and FBs from the same bronchoscopy examinations.

## 2. Methods

### 2.1. Patient Selection

Routine surveillance bronchoscopies were performed 1, 2, 4, 6, and 12 months following LTx, and thereafter when clinically indicated, to assess patients for ACR. Bronchoscopies performed in suspected ACR cases, hereafter referred to as bronchoscopies indicated for clinical reasons, were performed in symptomatic LTRs, LTRs with abnormalities in chest computed tomography, and LTRs with declining pulmonary function. Previously, FB frequently did not generate sufficient lung specimens to detect acute cellular rejection. Consequently, our lung transplant center changed the routine sampling procedure to include both FB and CB for bronchoscopies in an attempt to improve the diagnosis of ACR. All LTRs who underwent bronchoscopies at the University Hospital Zurich between February 2020 and April 2021 were evaluated in this retrospective study. FB and CB were obtained consecutively during the same bronchoscopy session. Exclusion criteria were age below 18 years, a lack of consent for the procedures, and dual antiplatelet therapy or anticoagulation therapy that could not be paused. All patient-related data, including demographic, clinical, and pathology data, as well as reports of bronchoscopy and spirometry, were obtained from electronic patient records.

### 2.2. Bronchoscopy and Periinterventional Care

All bronchoscopies were performed as in-patient procedures. Empiric intravenous antibiotic treatment was initiated on the day prior to bronchoscopy and continued until discharge on the day after bronchoscopy. The bronchoscopy was not performed if the laboratory work-up on the day of admission showed leukopenia, neutropenia, or increased inflammatory parameters (i.e., C-reactive protein or leukocytosis); the body temperature was more than 38.4 °C; or thrombocytes were <100 G/L. Vitamin K-antagonist anticoagulants were discontinued 5 to 7 days before the procedure. LTRs with an INR > 1.3 received vitamin K so that the INR was ≤1.3 on the day of the bronchoscopy.

All procedures were performed under local anesthesia and moderate propofol sedation using flexible Olympus (Olympus, Tokyo, Japan) bronchoscopes (190 series), which were inserted through an uncuffed tracheal tube (BronchoFlex, Rüsch, inner diameter 7.5 mm, Fellbach, Germany) to prevent laryngeal injury during extraction of CB samples. Bronchoscopies were initiated with a standardized BAL with 150–200 mL of normal saline, followed by FB and, subsequently, CB. Routinely, both biopsy procedures were performed during the same session, each according to official recommendations. ^19−24^ FB and CB were obtained unilaterally in different segments. Once wedged in position, a fluoroscope was used to guide the forceps and cryoprobe (Erbe 2.4 mm or 1.7 mm, Elektromedizin GmbH, Tübingen, Germany), which were advanced to the pleura and then retracted by 2 to 3 cm. For FB, anterior and posterior segments of the lower lobes were preferred, whereas CB was taken from the lateral basal bronchus and one of the segmental bronchi of the upper lobe; in total, five specimens were collected by FB and another two were collected by CB. A freezing time of four to five seconds (2.4 mm cryoprobe) or six to seven seconds (1.7 mm cryoprobe) was applied before the bronchoscope together with the cryoprobe were removed en bloc. Xylometazolin (2 mg) and tranexamic acid (500 mg) were prepared to be instilled over the working channel of the bronchoscope in cases of moderate to severe bleeding. In addition, a bronchial blocker (Rüsch, size Ch.6) was always ready to use but not prophylactically installed. After the CB sample was thawed in normal saline at room temperature, it was directly transferred to the formalin solution. FB samples were put directly into the formalin solution. Every procedure was performed by respiratory physicians experienced in CB with a minimum of 20 procedures per year.

### 2.3. Outcomes

The primary endpoint of this retrospective study was to compare the diagnostic yield of FB with that of CB for the diagnosis of ACR in LTRs. The secondary endpoints were the impact on treatment decisions and procedure-related morbidity. The biopsy samples were scored for ACR according to the ISHLT criteria (A0–A4) by one dedicated transplant pathologist at University Hospital Zurich (GA) [9]. For the purpose of the study, two independent, dedicated lung pathologists (SA and VB) additionally reviewed histological samples. They assessed FB and then CB separately without knowledge of the other pathologists’ assessments. Furthermore, SA and VB were blinded to patient data and follow-up.

In cases of ACR grade A1, the prednisolone dosage was increased by 10 mg/day for five days. In cases of grade A2 rejection, intravenous steroid pulse therapy with methylprednisolone 125 mg/day (1–2 mg/kg/day) for three days was given. In cases of persistent grade A2 or A3 rejection, the methylprednisolone pulse dose (10–15 mg/kg/day) was increased to 0.5 or 1 g/day over 3 days, and the calcineurin inhibitor was switched. Since CB is not a routine sampling practice for the detection of ACR according to the ISHLT guidelines, the severity of bleeding was assessed with the Nashville Bleeding Scale in our clinical practice [28]. Grade 1 bleeding requires the suctioning of blood for less than one minute, whereas grade 2 bleeding requires suctioning for more than one minute and repeated wedging or instillation of vasoactive substances or thrombin, respectively. The premature interruption of the procedure, balloon blocker insertion for less than 20 min, or selective intubation is classified as grade 3 bleeding. Grade 4 bleeding is defined as selective intubation or balloon blocker insertion for longer than 20 min in addition to red blood cell transfusion, selective bronchial artery embolization, admission to the intensive care unit, surgical intervention, or resuscitation [28]. The grades of bleeding were reported in the patient files in our routine clinical practice.

We use bedside ultrasound in routine practice to check for pneumothorax after TBB in all patients. Pneumothorax was screened with immediate bedside chest ultrasonography and chest radiography in an upright position two hours after the procedure.

### 2.4. Statistical Analysis

Quantitative data are presented as means ± standard deviation (SD). Categorical variables are expressed as frequencies (n) and percentages (%). The degree of interobserver agreement was determined by calculating the kappa index (KI): poor agreement, KI = 0; slight agreement, KI = 0.01–0.20; fair agreement, KI = 0.21–0.40; moderate agreement, KI = 0.41–0.60; good agreement, KI = 0.61–0.80; and excellent agreement, KI = 0.81–1.00. Data were analyzed using SPSS software, version 26.0 (IBM, New York, NY, USA).

Approval from the Competent Ethics Committee was waived because of formal non-objection (BASEC-ID 2020-01868).

## 3. Results

A total of 320 macroscopic biopsy samples (202 by FB and 118 by CB) were obtained during 63 procedures in 35 LTRs. The mean numbers of CBs and FBs per procedure were 1.87 (±0.6) and 3.21 (±1.2), respectively. The median time from LTx to bronchoscopy was 211 days (IQR 129–389 days). Of the procedures, 46 were routine surveillance bronchoscopies and 17 were bronchoscopies indicated for clinical reasons. The demographic and clinical characteristics are presented in Table 1.

**Figure 1:**
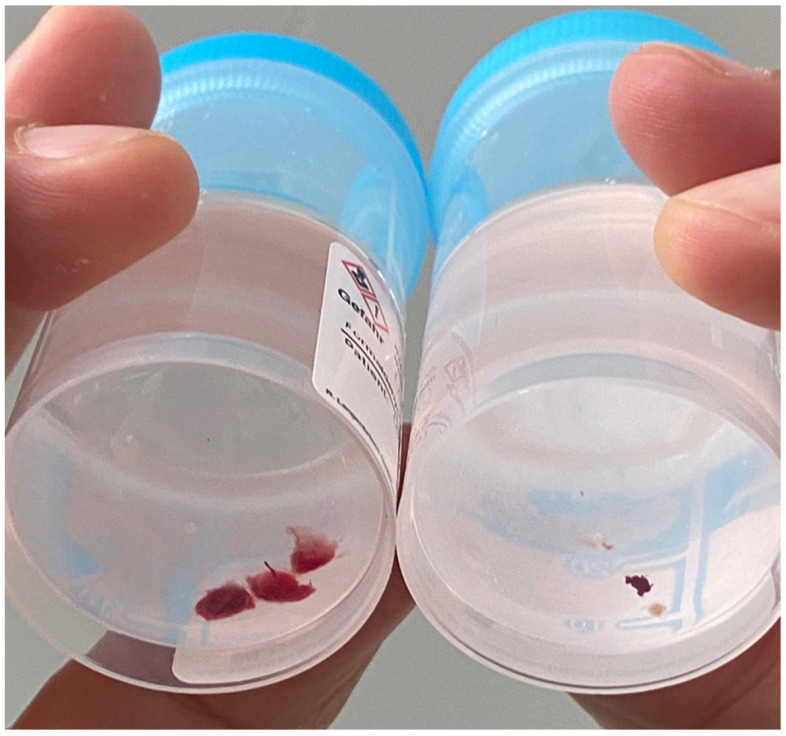
Macroscopic view of specimens obtained with cryobiopsy (left side) and forceps biopsy (right side).

**Figure 2:**
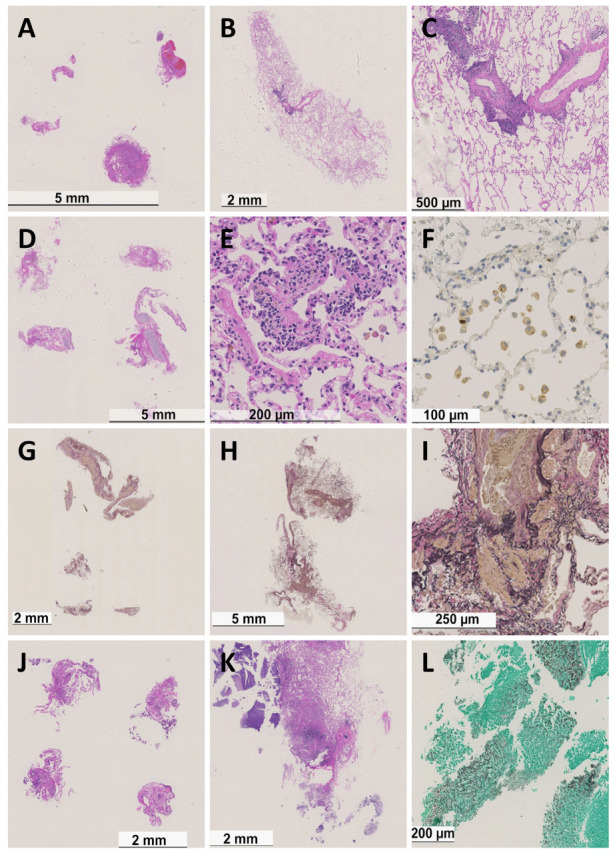
Histology of transbronchial biopsy specimens. (**A**,**D**,**G**,**J**) were obtained by FB. (**B**,**C**,**E**,**F**,**H**,**I**,**K**,**L**) were obtained by CB. Patient 1 (**A**–**C**) showed moderate ACR, ISHLT Grade A3; patient 2 (**D**–**F**) showed mild ACR, ISHLT Grade A2, and cytomegalovirus infection; patient 3 (**G**–**I**) showed obliterative bronchiolitis, ISHLT C1, classified as chronic airway rejection; patient 4 (**J**–**L**) showed Aspergillus infection. (**A**–**E**), (**J**,**K**): hematoxylin–eosin, (**F**): immunohistochemistry for CMV, (**G**–**I**): Elastica van Gieson staining, (**L**): Grocott staining.

The histological results obtained by CB and FB are shown in Table 2. The mean numbers of lung specimens obtained by CB and FB were 1.9 ± 0.6 and 3.2 ± 1.2, respectively. The larger sizes of CB samples compared with FB samples (Figure 1) enabled a definitive diagnosis in 95.2% of patients compared with 9.5% of patients by FB. Consequently, a high number of specimens obtained by FB were non-diagnostic because of the small specimen size of the biopsies and because our protocol obtained only five specimens by FB. According to the recent ISHLT guidelines, at least five pieces of well-expanded lung parenchyma are required for an assessment of ACR [9]. ACR (A1–A3, minimal–moderate acute rejection) was detected in 18 cases (28.6%) by CB, resulting in a change of immunosuppressive therapy, and only three cases of ACR (4.8%) were detected by FB. CB also revealed the diagnosis of bronchiolitis obliterans in two cases, with CMV and Aspergillus infection in one case each, whereas FB did not detect CMV or Aspergillus infection (Figure 2). There was no A3 rejection detected by FB, whereas one case was detected by CB (Table 2 and Table 3).

The interobserver agreement between the three pathologists for FB compared with that of CB was moderate (KI = 0.54). The highest agreement was observed among pathologists 2 and 3 (P2 and P3), with good agreement for FB compared with CB (KI = 0.69 and 0.67) (Table 4).

No major complications occurred despite obtaining FB and CB during the same session. Moderate (grade 2) and severe (grade 3) bleeding were observed in 23 patients (36.5%) and one patient (1.6%), respectively. One patient was diagnosed with a subsegmental pulmonary embolism on the contralateral side 15 days after bronchoscopy, which seemed to be unrelated to the bronchoscopy procedures.

**Figure 3:**
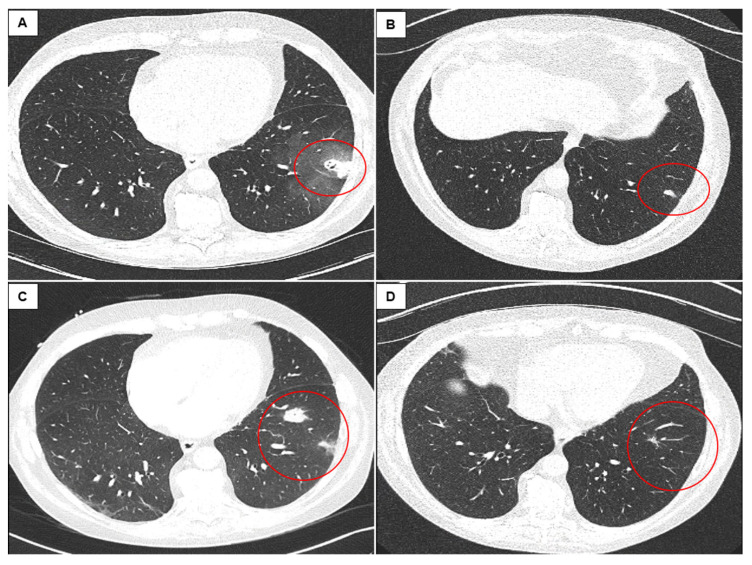
Computed tomography of the chest performed 20 (**A**) and 89 days (**B**) after cryobiopsy and forceps biopsy in patient 1 and 16 (**C**) and 94 days (**D**) after cryobiopsy and forceps biopsy in patient 2, demonstrating slowly disappearing subsolid and solid consolidations (red circles).

Pneumothorax occurred in four (6.3%) cases requiring chest tube placement, one of which was unrelated to the biopsy, as it occurred after central venous catheter placement. It was not possible to attribute pneumothorax to CB or FB because of the serial application of both procedures during the same bronchoscopy session. Furthermore, subsolid and solid consolidations were observed after bronchoscopy in two patients who had computed tomography of the chest for other reasons. All bleeding lesions decreased in size three months later, suggesting that localized bleeding was the most probable cause (Figure 3).

## 4. Discussion

In this retrospective study, FB was compared with CB; both were performed consecutively in LTRs during the same procedure. CB detected ACR in 18 patients (28.6%), providing an improved diagnostic yield for ACR diagnosis compared with FB, which detected ACR in three patients (4.8%); CB also had an acceptable safety profile, resulting in reclassification and changes in treatment strategy in one-third of cases.

In a study by Takizawa et al., surveillance bronchoscopies with specimens obtained by FB in asymptomatic LTRs with stabile lung function enabled a change in treatment in 7.7% of the cases [29]. Similarly, we found that alterations in the treatment regimens in asymptomatic LTRs were indicated in three patients (6.5%) (ACR A1 and A2: 2.2% and 4.3%) when treatment decisions were based on FB results only (Table S1 of the Supplement). On the other hand, treatment change after the diagnosis of ACR by CB was required in 11 (24%) patients (ACR A1, A2, and A3: 10.9%, 10.9%, and 2.2%) in asymptomatic LTRs (Table S1 of the Supplementary Materials). In the whole cohort (including those with bronchoscopies indicated for clinical reasons), treatment changes were necessary for 28.6% and 4.8% of patients in CB and FB, respectively. In addition, CB provided the diagnosis of ACR grade 3 in one patient with histologically proven CMV and Aspergillus infection, while FB and BAL revealed negative findings. Therefore, we assume that relevant diagnoses in LTRs, such as asymptomatic ACR as an important risk factor for the development of CLAD or infections with opportunistic pathogens, are likely to be missed when only FB is performed [1].

A further question concerning the indication for bronchoscopies in LTRs is whether they should be performed as scheduled surveillance bronchoscopies in asymptomatic LTRs or whether they should be performed for clinical reasons only. Valentine et al. found no statistical differences in ACR diagnosis, 1- and 3-year BOS-free survival and patient survival, or infection rates when comparing bronchoscopies for surveillance purposes with those performed only when clinically indicated [30]. Of the 46 surveillance bronchoscopies we performed in asymptomatic LTRs, ACR was found by CB in 11 (24%) LTRs, which confirms the importance of surveillance bronchoscopy in asymptomatic LTRs (Table S1 of the Supplement). Furthermore, 1- and 3-year BOS-free survival is a limited follow-up period since 60% of LTRs live longer than 3 years [31].

Although we used CB and FB consecutively during the same procedure, the bleeding and pneumothorax rates in the present study were relatively low and comparable to those reported in previous publications on CB in LTRs (Table 5) [18,19,20,22,23,24,29]. 

The reported adverse event rate after FB is slightly lower than that in the current study (6%), with a pneumothorax rate of 0% to 5% and a bleeding rate between 1% and 52% [21,22,23,25,26,27,32]. However, CB in LTRs might have unusual consequences, as we found new pulmonary lesions in the computed tomography scans of two patients in which TBBs by CB were taken. For example, one of these patients presented with a cavitated subpleural lesion (Figure 3). Similarly, in the prospective study of Loor et al., up to 91% of lung allografts demonstrated new opacities after TBB, some of them persisting for more than six months [32]. Thus, in LTRs, altered reparative mechanisms after TBB, in particular after CB, seem to exist and may lead to new and unusual radiological findings.

There are several limitations in our study, which are primarily due to its retrospective study design and the single-center experience. Since no other lung transplant centers were included in our study, we could not analyze whether our histological results and complications were correlated with those of other transplant centers. We could not compare the complications between FB and CB since both procedures were obtained consecutively during the same bronchoscopy session. Moreover, we did not reach the target number of five good FB samples required by the recent ISHLT guidelines because some samples did not show well-expanded lung parenchyma. Furthermore, our results show a moderate interobserver agreement between all three independent pathologists. However, despite these drawbacks, the study also has several strengths. First, this is the first study with matched evaluation of both FB and CB in LTRs. Thus, both FB and CB were available for each patient, allowing us to confine the study to a relatively small sample size. Second, all procedures were performed by three (CS, FG, and DF) experienced respiratory physicians trained in CB. Thus, a learning curve can be excluded. Third, histology specimens were independently assessed by three dedicated lung pathologists. Nevertheless, further randomized prospective studies and long-term follow-up are needed to endorse the efficacy and safety of CB after LTx.

## 5. Conclusions

In summary, CB provided an improved diagnostic yield of ACR diagnosis, leading to reclassification and changes in treatment strategy in 28.6% of cases. Prospective studies are needed to define the role of CB in post-LTx surveillance. Interobserver disagreement between different pathologists needs to be considered.

## Figures and Tables

**Table 1:** Demographic and clinical parameters of the study cohort.

	Total (N = 63)
Female sex	28 (44.4)
Age	56.4 ± 8.83
Surveillance bronchoscopy	46 (73)
Days from LTx to bronchoscopy	211 (IQR 129–389)
Pulmonary diseases n	
COPD	40 (63.5)
CF	5 (7.9)
Non-CF	3 (4.8)
Vaskulitis	2 (3.2)
IPF	2 (3.2)
NSIP	5 (7.9)
EAA	5 (7.9)
COP	1 (1.6)
Biopsy side right/left	31/32 (49.2/50.8)
Side of LTX bilateral/unilateral	59/4 (93.7/6.3)
Previous anticoagulation drug treatment	17 (27)
Platelet aggregation inhibitor treatment	7 (11.1)
FEV1 (liters and percent predicted)	2.5 ± 0.9; 81.9 ± 21.1
Diffusion capacity, percent predicted	71.9 ± 22.7
Thrombocytes count, µl	277.3 ± 100.8
INR	1.0 ± 0.1

Values are displayed as n (%) or mean ± SD. FEV1% predicted value was calculated according to the recommendations of the Global Lung Function Initiative. Abbreviations: LTx, lung transplantation; CF, cystic fibrosis; non-CF bronchiectasis (non-cystic fibrosis bronchiectasis); IPF, idiopathic pulmonary fibrosis; NSIP, nonspecific interstitial pneumonia; EAA, exogen allergic alveolitis; COP, cryptogenic organizing pneumonia.

**Table 2:** Results and complications.

	CB	FB
Diagnostic yield for diagnosis of ACR %	28.6	4.8
Grade of ACR		
A0	45 (71.4)	60 (95.2)
A1	9 (14.3)	1 (1.6)
A2	8 (12.7)	2 (3.2)
A3	1 (1.6)	0
A4	0	0
Diagnostic yield of chronic rejection C1 %	3.2	3.2
Quality of TBB		
Diagnostic	60 (95.2)	6 (9.5)
Non-diagnostic	3 (4.8)	57 (90.5)
Specimen		
Size mm	10.1 ± 7.1	2.3 ± 1.8
Number	1.8 ± 0.6	3.2 ± 1.2
CMV Infection	1 (1.6)	0
Aspergillus infection	1 (1.6)	0
Bleeding Grade		
0	4 (6.3)	
1	35 (55.6)	
2	23 (36.5)	
3	1 (1.6)	
4	0	
Pneumothorax (%)	4 (6.3%)	

Values are displayed as n (%) or medians ± SD. Results were obtained according to the histologic results of pathologist 1 (P1). Abbreviations: ACR, acute cellular rejection; TBB, transbronchial biopsy; SD, standard deviation.

**Table 3:** Distribution of the histological results by the three independent transplant pathologists for diagnosis of acute cellular rejection.

	Pathologists		
	P1	P2	P3
Forceps biopsy			
A0	60 (95.2)	57 (90.5)	55 (87.3)
A1	1 (1.6)	5 (7.9)	4 (7.9)
A2	2 (3.2)	1 (1.6)	3 (4.8)
Crybiopsy			
A0	45 (71.4)	42 (66.7)	45 (71.4)
A1	9 (14.3)	14 (22.2)	12 (19)
A2	8 (12.7)	6 (9.5)	5 (7.9)
A3	1 (1.6)	1 (1.6)	1 (1.6)

Values are presented as n (%). Abbreviations: P1–P3, Pathologists 1–3.

**Table 4:** Degree of interobserver agreement for the forceps biopsy and cryobiopsy as determined by the kappa index (range 0–1.00).

	Forceps Biopsy	Cryobiopsy
All 3 P	0.54	0.54
P1 vs P2	0.54	0.54
P1 vs P3	0.43	0.58
P2 vs P3	0.69	0.67

Poor agreement, KI = 0; slight agreement, KI = 0.01–0.20; fair agreement, KI = 0.21–0.40; moderate agreement, KI = 0.41–0.60; good agreement, KI = 0.61–0.80; and excellent agreement, KI = 0.81–1.00. Abbreviations: P1–P3, pathologists 1–3; KI, kappa index.

**Table 5:** Reported complications after cryobiopsy in lung transplant recipients.

Complications after Cryobiopsy in Lung Transplants			
Author, Year	Bleeding	Pneumothorax	No. of Procedures
Fruchter, 2013 [23]	1 (3)	0 (0)	40
Yarmus, 2013 [22]	12 (57)	1 (5)	21
Roden, 2016 [20]	5 (19)	1 (4)	27
Gershman, 2018 [19]	5 (3)	9 (5)	201
Loor, 2019 [29]	24 (8)	25 (8)	321
Mohamed, 2020 [18]	6 (8)	1(1)	75
Montero, 2018 [24]	9 (23)	5 (13)	40

Values are presented as N (%). No., Number.

## Data Availability

The data that support the findings of this study are available on request from the corresponding author, C.S.

## Supplementary Materials

The following supporting information can be downloaded at: https://www.mdpi.com/article/10.3390/life12060898/s1, Table S1: Grade of acute cellular rejection in surveillance bronchoscopy and clinically indicated bronchoscopy.
