# Supplementary material for: Transbronchial Cryobiopsy Compared to Forceps Biopsy for Diagnosis of Acute Cellular Rejection in Lung Transplants: Analysis of 63 Consecutive Procedures
Source: Life (Basel). 2022 Jun 15;12(6):898. doi: 10.3390/life12060898 (PMC9225122; doi:10.3390/life12060898)
Supplement: Supplementary file 1 [file life-12-00898-s001.zip › life-1705507-supplementary.pdf]

Supplementary Material

Table S1: Grade of acute cellular rejection in surveillance bronchoscopy and clinically indicated bronchoscopy

	CB	FB
Surveillance bronchoscopy	46	46
A0	35 (76)	43 (93.5)
A1	5 (10.9)	1 (2.2)
A2	5 (10.9)	2 (4.3)
A3	1 (2.2)	0
Clinically indicated bronchoscopy	17	17
A0	9 (53)	16 (94.1)
A1	4 (23.5)	0
A2	4 (23.5)	1 (5.9)
